# *Salmonella enterica* frequency in backyard chickens in Vermont and biosecurity knowledge and practices of owners

**DOI:** 10.3389/fvets.2022.979548

**Published:** 2022-09-22

**Authors:** Katalin M. Larsen, Melissa DeCicco, Katherine Hood, Andrea J. Etter

**Affiliations:** ^1^Etter Laboratory, Nutrition and Food Sciences Department, The University of Vermont, Burlington, VT, United States; ^2^Microbiology and Molecular Genetics Department, The University of Vermont, Burlington, VT, United States

**Keywords:** backyard chickens, *Salmonella enterica*, biosecurity, animal husbandry, poultry (chicken), homesteading, food safety

## Abstract

The popularity of backyard chickens has been growing steadily over the past 10 years, with Covid-19 stay at home orders in 2020 yielding an added boost in popularity. Concurrently, cases of salmonellosis from live poultry exposure have also risen. Previous research on backyard chicken owners has focused primarily on urban chicken owners, which may have differing knowledge and biosecurity habits from rural backyard chicken owners. The goal of this study was to investigate the prevalence of *S. enterica* in rural and urban flocks of chickens in the state of Vermont and to determine what attitudes toward and knowledge about *S. enterica* owners had, as well as what biosecurity practices they used. We conducted two surveys in Vermont between 2019–2022; a pilot study tied to sampling for *Salmonella enterica* in backyard chicken flocks from 2019–2021 and a statewide study in 2022 to determine the prevalence of backyard chickens in Vermont and obtain representative survey data from backyard chicken owners. We found (i) overall, 19% (8/42) backyard chicken flocks from 2019–2021 had *S. enterica*, but *S. enterica* rates varied substantially by year; (ii) backyard chicken owners were wealthier and more educated than the average Vermonter and generally lived in rural areas; (iii) participants in the statewide survey had much lower uptake of good biosecurity habits compared to the pilot survey; (iv) despite increased messaging about backyard chicken-associated salmonellosis and good biosecurity measures over the past several years, uptake of biosecurity measures is inconsistent, and rates of unsafe practices such as kissing or cuddling chickens have increased in Vermont. Overall, the data indicate the need for improved messaging on biosecurity and risks associated with backyard chickens

## Introduction

Researchers estimate that more than one million people contract non-typhoidal *Salmonella enterica* in the U.S. each year, leading to an estimated $3.7 billion annually in lost wages, productivity, healthcare costs, and mortality (1, 2). Salmonellosis is most frequently acquired through eating contaminated meat (especially poultry), and eggs (3). However, salmonellosis can also be contracted *via* contact with live reptiles, including turtles, and backyard poultry (4, 5). These zoonotic infections represent roughly 11–20% of all *S. enterica* infections each year in the United States, and cause 51.2% of salmonellosis cases in children under 10 years old (4, 5). Additionally, across all ages, animal-associated salmonellosis has higher odds of hospitalization compared to food-associated salmonellosis (5). Finally, over the past 10 years, the rate of live poultry-associated salmonellosis rose to nearly 1,800 cases per year in 2020 (6), with the CDC reporting in 2020 that there had been 77 outbreaks of *S. enterica* since 2010 (7).

Despite this rise in salmonellosis associated with live poultry, only a small number of studies have been performed to determine the prevalence of *S. enterica* in backyard chickens in the U.S and the biosecurity practices associated with the presence or absence of *S. enterica* in flocks. A study of flock characteristics and owner biosecurity habits for backyard poultry flocks in Maryland in 2011 found that just 65.8% of owners consistently washed their hands after interacting with their flock, and even fewer (31.7%) had dedicated footwear for the poultry pens (8). Additionally, nearly half (44%) of flocks were free-ranged, and owners reported their flocks had interactions with multiple other species, including wild birds (53.7% of flocks), pets (75.6%), livestock (31.7%), wild carnivores (46.3%), and rodents (36.6%) (8). Sixty-one percentage of owners had had birds for fewer than 5 years, and 17.1% of owners had had birds for less than a year, indicating a potential increase in the popularity of backyard chickens (8). The study did not find any *S. enterica* in the 39 flocks they evaluated (8).

Researchers in Colorado in 2012 surveyed 807 backyard flock owners on flock characteristics, housing, health, and the owners' biosecurity practices (9). Most flocks contained under 50 birds, and most flocks (59.9%) were housed in an outdoor coop with fenced-in outdoor access (9). Owners typically washed their hands after handling birds (79%), but only 20% changed shoes after being around birds (9). Additionally, roughly 60% of owners quarantined new birds before introducing them to the flock (9). The researchers did not test for pathogens.

A study of backyard chickens in the greater Boston urban area from 2016 to 2017 tested 53 flocks and found that just one had *S. enterica* (10). Further, the *S. enterica* found was *Salmonella* Kentucky, which is rarely implicated in human illnesses, and therefore posed little risk to the owners (11). The researchers surveyed 30 of the owners on biosecurity habits and attitudes and found that 95.6% of families in the study who had children considered their birds to be pets, and 68.9% (20/30) of children reportedly petted the birds or picked them up (10). This suggested that owners and their children would have a high risk of contracting salmonellosis if their birds were infected with *S. enterica*.

A survey and observational (video) study of backyard poultry owners in Seattle in 2014 also observed poor biosecurity habits, including kissing and snuggling birds (12). Ultimately, 25% of participants indicated that they snuggled, kissed, touched their mouth, or ate/drank around their birds, while video recordings showed >50% of participants touched their face, and an additional 22% were recorded snuggling birds (12). However, that study did not assess *S. enterica* prevalence in the chickens.

A later study in Washington State in 2016 found that 1/34 flocks in counties around Seattle had *S. enterica* (13). The serovar found in the infected flock was I 4,[5],12; i-, a serovar frequently implicated in human illnesses (13–15). They also found that 83% of the *Escherichia coli* isolated from the flocks was resistant to ≥3 classes of antibiotics (13). Finally, the study found that 62% of owners (21/34) contained elderly or young family members who had direct contact with birds, and that 6% of their owners considered their poultry pets (13).

Despite these studies, numerous knowledge gaps remain. Kauber and McDonaugh's studies looked only at urban contexts (10, 12), while Shah's study included some rural farms, but did not survey owners on biosecurity (13). Meanwhile, Madsen's study was the only one to note the number of backyard chicken flocks in the state it was performed in, as Maryland at the time had a required backyard flock registry (8, 16). Perhaps most importantly, none of the studies was conducted over more than 12 months. The goal of this study was to investigate the prevalence of *S. enterica* in rural and urban flocks of chickens in the state of Vermont and to determine what attitudes toward and knowledge about *S. enterica* owners had, as well as what biosecurity practices they used.

## Methods

Vermont is a small state in the northeastern United States, with a high rural population [61.3% (17)] and a high prevalence of home food production (18). It experiences relatively cold winters and temperate, short summers, and is in zones 3b-5b (19, 20). In Vermont from 2016–2020, 20.8% of all reported salmonellosis cases were connected with live poultry, out of an average of 102 salmonellosis yearly cases (21). Importantly, children under 10 make up 30.2% of all live poultry associated salmonellosis patients in Vermont, despite making up only 20.5% of the population (4, 21, 22). To determine the rates of *S. enterica* in backyard chickens in Vermont and the biosecurity knowledge and practices of their owners, we conducted two surveys.

### Pilot survey

In this survey, owners of backyard chickens in Vermont who were willing to have their flocks tested for *S. enterica* were asked to complete a survey about their knowledge of *S. enterica* and food safety as well as their husbandry practices and some demographic questions (Supplementary Table S1). This survey was a sample of convenience and was completed by 43 backyard chicken owners from 2019 to 2021. Surveyed flocks were primarily located in Chittenden County, Vermont (Northwestern Vermont), and were recruited through advertisements in feed stores, on Facebook poultry fancier/homesteading pages, and through posts in Front Porch Forum (a neighborhood forum/newsletter site). Due to the fact there were no reliable numbers on backyard chicken ownership in Vermont, we simply strove to get at least 30–50 flocks, in line with previously published studies on backyard poultry flocks (10, 13). The survey consisted of three basic sections; questions about the owners' flock, including number of birds, breeds, other animals present, and housing/feed; questions on owners' biosecurity and egg handling habits; and questions about perceptions of risk. This survey was deemed exempt by UVM's Institutional Review Board (Approval #: STUDY00000237).

### Statewide survey

To obtain statewide, representative data, we contracted with UVM's Center for Rural Studies to field a statewide survey designed to determine the percent of Vermonters who keep backyard chickens as well as the habits of backyard chicken owners (Supplementary Table S2). It consisted of four types of question; questions about the owners' flock, including number of birds, breeds, other animals present, and housing/feed; questions on owners' biosecurity habits; questions about perceptions of risk., and demographic questions. This survey was approved by UVM's IRB as a modification of STUDY00000237.

#### Salmonella sampling

##### Cloacal swabbing

Cloacal swabs were taken from each bird and tested for the presence of *S. enterica* (23). Prior to starting sampling, our cloacal swab procedure was approved by UVM's IUCAC board (IACUC protocol 19-053). Data on each chicken's breed was recorded along with farm and swab number. A sterile swab was inserted into the cloaca of the bird, to collect fecal matter, returned to a sterile, labeled test tube, and brought back to the laboratory for testing using standard *Salmonella* enrichment protocols (23), with pre-enrichment in buffered peptone water (BD Difco, Franklin Lakes, NJ or Thermo Scientific, Waltham, MA) for 24 h, followed by sub-culturing into tetrathionate and Rappaport-Vasiliadis Broth (BD Difco, Franklin Lakes, NJ) at 37 and 42°C, respectively for 24 h. Tetrathionate and Rappaport-Vasiliadis enrichments were then streaked onto xylose lysine tergitol_4_ agar (BD Difco, Franklin Lakes, NJ) and incubated at 37°C for at least 24 h. After 2019, XLT4 plates were incubated for 48 h, to better capture slow-growing samples.

##### Bedding sampling

A roughly quart-sized sample of soiled bedding was collected, either by the owners or by our team and placed in a clean Ziploc bag or container. The sample was brought back to the laboratory and frozen at −20 or −80°C if it could not be processed immediately. To detect *S. enterica*, a 25 gram sample was weighed out aseptically into a stomacher bag. Hundred milliliter of Buffered Peptone Water (BD Difco, Franklin Lakes, NJ) was added and the sample stomached (Seward, West Sussex, UK) for 1 min in on the standard stomaching speed or hand-massaged for 2 min. Samples were pre-enriched at 37°C for 4 h to allow for bacterial recovery before proceeding with the *Salmonella* detection protocol above.

##### Salmonella confirmation

Presumptive positive colonies from XLT4 (black, pink, or colorless on a red background) were streaked for isolation and screened with PCR for the *hilA* gene (24). We developed our own primers, as the primers in Pathmathan et al., did not work well for us. Primer sequences: *hilA_FW_2* 5' GGA CAG GGC TAT CGG TTT AAT 3' and *hilA_RV_2* 5' CAA ACT CCC GAC GAT GTA TTC T 3'. DNA for PCR was obtained by boiling a single colony in 100 μl of dd_H_20 and centrifuging to pellet cell debris. PCR was performed using GoTaq Colorless 2X master mix (Promega, Madison, WI) in a BioRad T100 thermal cycler (BioRad, Hercules, CA), and results visualized *via* a 1% agarose gel. PCR cycling conditions were as follows: 95°C for 5 min, followed by 30 cycles of 95°C for 1 min, 50°C for 1 min, 72°C for 1 min. A final extension was performed at 72°C for 5 min before cooling to 12°C.

##### Statistics

Chi-squared analysis was used to determine statistically significant differences between the pilot and statewide surveys and among groups within the survey, with a Fisher's Exact Test for samples with ≤ 4 in a category. Statistics were run in Rstudio (version 3.5.2) or in SPSS (version 1.0.01275).

## Results

We conducted two surveys. The first was a survey of backyard chicken owners in Vermont who agreed to cloacal swabbing or bedding sampling of their flocks from 2019 to 2021 (hereafter referred to as the “pilot survey”). The second was a large survey of Vermonters conducted by the UVM Center for Rural Studies, designed to determine the percentage of Vermont residents with backyard chickens and the knowledge and biosecurity habits of backyard chicken owners in Vermont (hereafter referred to as “Statewide survey”).

### Pilot survey

Our pilot survey yielded 43 respondents from 2019 to 2021. The mean number of birds in each flock was 10, while the median number was eight (Table 1). However, flock size ranged from 2 to 75 birds. Most (30/43) purchased at least some birds sourced from a commercial hatchery, either directly or through a feed store. A quarter (11/43) had also acquired birds from a friend, acquaintance, the humane society, or *via* chicken swaps. Two owners had also purchased birds at fairs, and five owners reported hatching chicks from their own flocks. The most popular breeds were Orpingtons [mostly Buff Orpingtons, with some other colors (23/43)], Americauna (22/43), and Barred Rocks (16/43), followed by Rhode Island Reds (15/43) and Wyandottes (14/43).

**Table 1:** Characteristics of flocks of backyard chickens in Vermont.

**Number of chickens per flock**	**Pilot survey**	**Statewide survey**
Mean	10.37	19.5
Median	8	10
Mode	10	6
Range	2 birds-75 birds	–
Source	Percent of flocks from source (Number/total)	
Friend/acquaintance	25.6% (11/43)	–^b^
Commercial hatchery	69.8% (30/43)	–
Hatched from own flock	11.6% (5/43)	–
Fair	4.7% (2/43)	–
Housing source	Percent of flocks with housing (Number/total)	
Penned at least part time	61.4% (29/43)	49.5% (191/386)
Free range at least part time	37.2% (16/43)	46.6% (180/386)
Penned in moving area (e.g., mobile chicken unit)	9.3% (4/43)	6.7% (26/386)
Inside only	–	4.1% (16/386)
Feed	Percent of flocks (Number/total)	
Commercial feed	100% (43/43)	93.5% (375/401)
Table scraps/food scraps	65.1% (28/43)	73.3% (294/401)
Forage	58.1% (25/43)	72.3% (290/401)
Other	–	5.0% (20/401)
Other animals present	Percent of flocks with species (Number/total)	
Any other animal	86.0% (37/43)	-
Dogs	72.1% (31/43)	67.8% (272/401)
Cats	48.8% (21/43)	49.1% (197/401)
Goats	11.6% (5/43)	9.5% (38/401)
Horses	9.3% (4/43)	11.2% (45/401)
Cattle	–	6.2% (25/401)
Rabbits	4.7% (2/43)	4.5% (18/401)
Llamas/alpacas	2.3% (1/43)	0.5% (2/401)
Sheep	2.3% (1/43)	5.5% (22/401)
Pigs	–	5.5% (22/401)
Other poultry	2.3% (1/43)	16.2% (65/401)

^a^Question not included in survey.

Most flocks (29/43) were penned in a fixed area around their coop at least some of the time, while 37.2% of flocks (16/43) were free ranged at least part of the time, and four flocks used a form of mobile chicken unit. All respondents fed at least some commercial feed to their birds, though most supplemented with table scraps (28/43) or forage (25/43). Most flocks (86%; 37/43) had at least one other species of domestic animal present on site, with dogs (31/43) and cats (21/43) being by far the most common. Four owners also had horses, and five had goats (2/5 farms with horses and chickens also had goats). Two farms had rabbits, one farm had llamas, and one farm had sheep. However, it was unclear how much interaction occurred between domestic animals.

We asked owners whether their flocks might have contact with wildlife. Most answers correlated with the housing situations the owners had described. However, in four cases, owners indicated that their chickens probably did not have exposure to wildlife, despite indicating the birds were free ranged at least part of the time, and six owners said their birds definitely had exposure to wildlife, despite indicating the birds were penned without free-range access. These answers, while at first surprising, are understandable; the penned flocks may have previously experienced predation, leading to the “definitely yes” response. Conversely, the owners of the free-range birds who were certain their birds did not interact with wildlife may not think of wild birds as wildlife.

Motivations for keeping poultry varied (Table 2), but the most popular top three reasons for keeping poultry in 2019–2021 were that eggs from backyard chickens were tastier (28/43 responses), chickens were fun/pets (22/43 responses) and that backyard chicken eggs were healthier (25/43 responses). Sustainability came in fourth (19/43), followed by “A good experience for children” (14/34). Notably, for the five participants who chose “chickens are a good learning experience for children” as their top reason for keeping chickens, 4/5 chose “pets/companionship” as one of their other top three choices, indicating that for families with children, backyard chickens are frequently viewed as pets.

**Table 2:** Owner's motivations for keeping poultry.

	**Pilot survey**	**Statewide survey**
**Top reasons for keeping chickens** ^**a**^		
Eggs/meat tastier than store brought	65.1% (28/43)	76.1% (305/401)
Eggs healthier than store brought	58.1% (25/43	–^b^
Pets/companionship/fun	51.2% (22/43)	53.6% (215/401)
More sustainable	44.2% (19/43)	–
Good experience for kids	32.6% (14/43	–
Food independence	9.3% (4/43)	43.4% (174/401)
Pest control/bug control	7.0% (3/43)	31.9% (128/401)
Other	23.3% (10/43)	4.5% (18/43)

^a^For the pilot survey, we only assessed the top three reasons. The statewide survey was set up as “select any that apply”.

^b^Question not asked in survey.

Unsurprisingly, given the varying motivations, biosecurity practices varied widely (Table 3). Nearly all owners (90.7%; 39/43) said they avoided kissing their birds, while nearly as many (84.1%; 37/43) said they washed their hands after handling their chickens. In contrast, only 25/43 (58.1%) said they avoided snuggling their birds, and just 26/43 (60.5%) indicated they washed their hands after handling eggs. An additional 12/43 (27.9%) responded that they washed their hands only if the eggs were dirty. Only 22/43 (51.2%) of owners indicated they changed their shoes after walking around in the chicken area; an additional owner said they washed their shoes “if dirty from chicken area.” Finally, eight owners (18.6%) said they wore masks while cleaning their coops.

**Table 3:** Backyard chicken owners' biosecurity habits and knowledge of *Salmonella* risks.

**Survey question**	**Pilot survey**	**Statewide survey**
**Biosecurity habits**	
Wash hands after handling chickens	84.1% (37/43)	75.8% (304/401)
Wash hands after handling dirty eggs	88.4% (38/43)	74.8% (300/401)
Wash hands after handling eggs	60.5% (26/43)	68.6% (275/401)
Avoid kissing birds	90.7% (39/43)	47.4% (190/401)
Avoid snuggling birds (and touching)	58.1% (25/43)	36.4% (146/401)
Change shoes after walking in chicken area (and clothes)	51.2% (22/43)	42.6% (171/401)
Wear a mask when cleaning the chicken coop (and goggles)	18.6% (8/43)	31.2% (125/401)
Keep children from snuggling birds	37.2% (16/43)	21.4% (86/401)
Keep children from interacting with chickens	9.3% (4/43)	12.2% (49/401)
**Chickens can have** ***Salmonella*** **while appearing healthy**		
Yes	83.7% (36/43)	60.2% (228/379)
No	0.0%	2.6% (10/379)
Maybe/Don't know	16.2% (7/43)	37.2% (141/379)
**Likelihood of** ***Salmonella*** **infection in backyard chickens vs. commercial chickens**		
More	9.1% (4)	2.4% (9/377)
Less	65.1% (28/43)	51.2% (193/377)
No difference	25.6% (11/43)	13.8% (52/377)
Don't know	–^a^	32.6% (123/377)
**Likelihood of** ***Salmonella*** **in eggs from backyard chickens vs. commercial eggs**		
Higher	25.6% (11/43)	6.1% (23/378)
Lower	39.5% (17/43)	40.7% (154/378)
No difference	34.9% (15/43)	17.7% (67/378)
Don't know	–	35.4% (134/378)
**Likelihood of** ***S. enterica*** **in eggs from own chickens vs. commercial eggs/eggs from store**		
Higher	11.6% (5/43)	3.2% (12/378)
Lower	44.2% (19/43)	46% (174/378)
No difference	44.2% (19/43)	18.5% (70/378)
Don't know	–	32.3% (122/378)
***Salmonella*** **can be on inside of egg as well as outside**		
Yes/inside and outside	51.0% (22/43)	44.3% (167/377)
No/outside only	19.0% (8/43)	12.7% (48/377)
Unsure/I don't know	30.0% (13/43)	43% (162/377)

^a^Question not included in survey.

Last, we asked about interactions of children with the backyard flocks. Only 16/43 (37.2%) respondents said they kept children from snuggling birds, while just four (9.3%) respondents indicated they kept children from interacting with their flock. During 2021, we asked about the frequency of children interacting with the flocks. Of the nine surveys from 2021, 7/9 indicated that children “often” or “always” interacted with the birds. Only one of these respondents also indicated that they tried to keep children from snuggling birds, while one respondent who indicated children “sometimes” interacted with their flock also noted that they tried to keep children from snuggling chickens.

We asked a number of questions designed to provide insight into owners' knowledge and risk perception around backyard chickens and *Salmonella* risks (Table 3). All owners recognized that chickens could probably or definitely have *Salmonella* without seeming sick. We were curious whether backyard chicken owners thought backyard chickens were less likely to have *S. enterica* than commercial flocks. Most owners thought backyard chickens were either less likely (65.1%; 28/43) to have *S. enterica* than commercial chickens or that there was no difference (25.6%; 11/43). Just four owners (9.3%) said that backyard chickens might be more likely to have *S. enterica* than commercial chickens. Nearly half (48.8%; 21/43) of the respondents thought chickens purchased from commercial hatcheries might be more likely to have *S. enterica*, 41.9% (18/43) thought there was no difference, and 9.3% (4/44) thought the chickens might be less likely to have *S. enterica*.

We asked about the safety of eggs, both from backyard flocks in general, and from the respondent's backyard flocks. Respondents were more confident in the safety of their own chicken's eggs; 39.5% (17/43) of respondents thought backyard chickens' eggs were safer than commercial, while 44.2% (19/43) thought their own chickens' eggs were safer than commercial. The number of respondents indicating “No difference” increased from 15/43 (34.9%) when asked about backyard chicken eggs in general to 19/43 (44.2%) when asked about eggs from the respondents' own flocks. Further, the number of “Less safe” responses for backyard chicken eggs vs. commercial eggs decreased from 11/43 (25.6%) to 5/43 (11.6%) when asked about the safety of eggs from their own flock, indicating that owners have a strong bias toward the safety of their own flocks' eggs.

#### Salmonella prevalence in pilot survey

**Figure 1:**
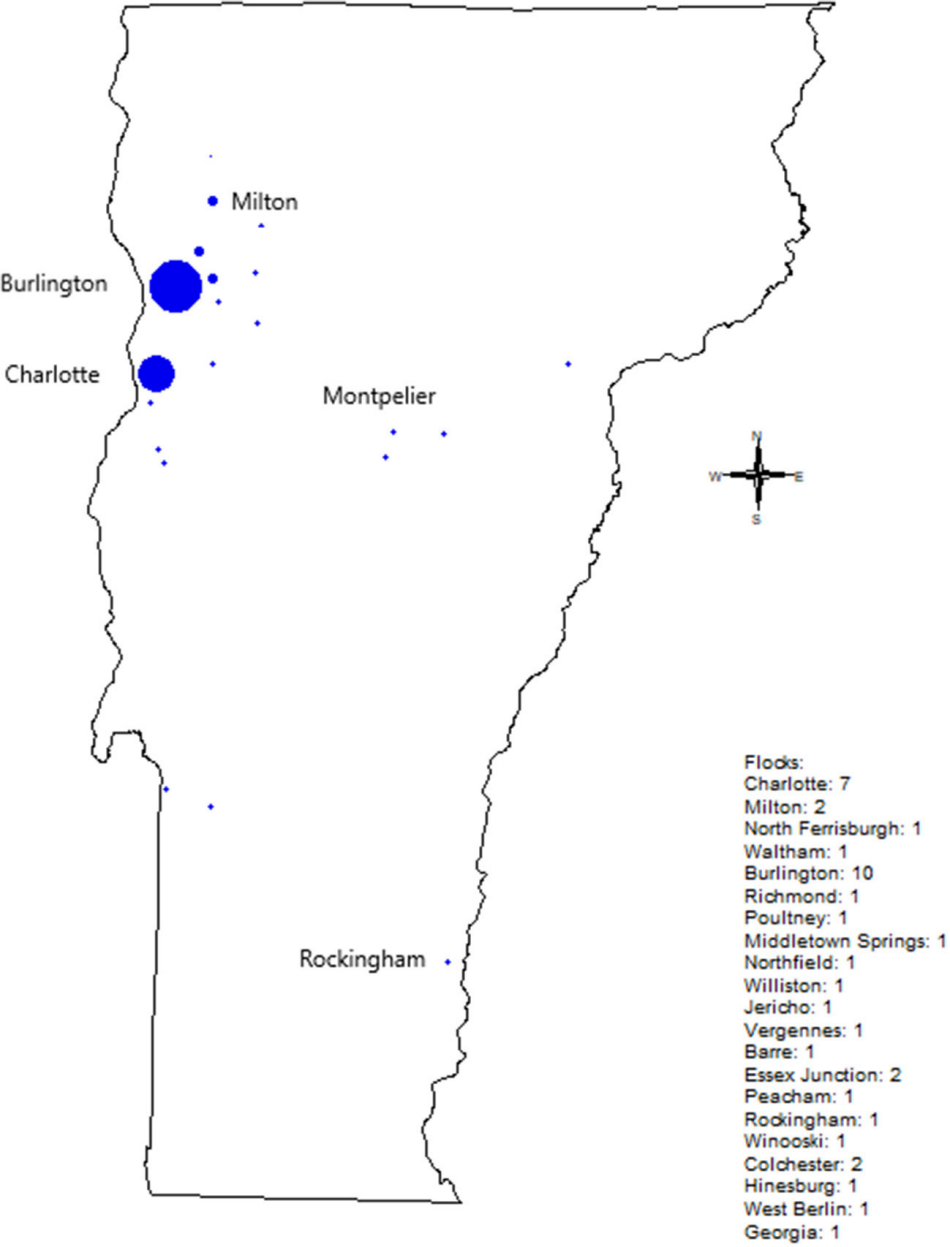
Backyard flocks sampled in Vermont by City. Circle size indicates the number of flocks tested, with numbers to the right of the figure.

During the pilot survey, we also conducted *Salmonella* sampling of the flocks, and continued after the end of the pilot survey. Flocks we sampled were concentrated near Burlington, Vermont, however we sampled flocks across most Vermont (Figure 1). Of the 42 flocks we sampled, eight tested positive for *S. enterica* (19%). During 2019, we sampled 28 flocks and found no *S. enterica*. In 2020, we sampled one flock, and found *S. enterica*. In 2021, we sampled 13 flocks, and found *S. enterica* in seven flocks (53.8% positive). Our sampling methods could have influenced *S. enterica* rates. During 2019-2020, we exclusively used cloacal swabs for sampling; in 2021, we transitioned to using a mix of cloacal swabs and bedding samples. Cloacal swabs are a less sensitive method of sampling than fecal or soiled bedding sampling (25); however, of the eight *S. enterica* positives, six were from cloacal swabs, so the increase in *S. enterica* rates is unlikely to be due to the move toward bedding samples.

*S. enterica* was slightly more common in rural flocks. While we had a mix of urban and rural flocks in all years we sampled, we found that 5/8 of the positive flocks were located in rural or semi-rural areas (we defined “semi-rural/urban” as a house in an urban area which backed up onto a large undeveloped/rural area or a cluster of houses/housing development in an otherwise rural area), and only three *S. enterica-*positive flocks were located in urban areas. In contrast, housing was not a major factor; 3/8 flocks with *S. enterica* were free-ranged at least some of the time, while 5/8 flocks with *S. enterica* were kept penned. We did not ask whether pens had roofs.

Finally, seven out of 43 owners surveyed in the pilot study indicated they had had diarrhea in the last year or since they'd gotten their birds, whichever was more recent, while four didn't remember, and the remainder had not had diarrhea since acquiring their birds/in the last year. Despite this being a common question in previous published surveys, we did not find it predictive. In the five surveyed flocks which we found had *S. enterica*, 3/5 owners responded that they had not had diarrhea, one couldn't remember, and only one indicated she'd had diarrhea in the past year/since getting her chickens.

#### Statewide survey

Because Vermont does not require owners to register their flocks, the number of backyard chickens in Vermont was unknown, and the representativeness of our pilot survey was unclear. To obtain statewide, representative data on the number of backyard chicken owners in Vermont and their knowledge and biosecurity practices, we contracted with UVM's Center for Rural Studies to conduct a large-scale survey using CRS databases of contact information.

The statewide survey yielded 1,730 responses. Of these, 401 (23.2%) reported having backyard chickens or having had a flock in the past year. This indicates there are likely nearly 150,000 backyard chicken owners in Vermont [2021 population of 645,570 (22)]. Backyard chicken owners in the CRS survey (Table 4) were primarily female (66.7%; 216/324), older than 35 (95.7%; 328/350) and living in rural areas (82.2%; 304/370). Most had completed a college degree, technical degree, or certificate (72%; 267/369), and 53.1% had a total yearly household income of $85,000 or more. 82.2% indicated they lived in a rural area, with just 8.4% indicating they lived in an urban area. Finally, just over 40% of backyard chicken owners reported children under 18 in the household (40.2%; 149/371).

**Table 4:** Demographic characteristics of backyard chicken owners.

	**Pilot survey**	**Statewide survey**
**Gender**		
Female	83.7% (36/43)^a^	66.7% (216/324)
Male	16.3% (7/43)	32.4 % (105/324)
Transgender/non-binary	–	0.9% (3/324)
**Age**		
18–24	–^b^	0.3 % (1/350)
25–34	–	6% (21/350)
35–44	–	17.1% (60/350)
45–54	–	23.4% (82/350)
55–64	–	26.6% (93/350)
65 or over	–	26.6% (93/350)
Median age		>65 years
**Household Income**		
$25,000 or less	–	5% (17/343)
$25,000–45,000	–	11.7% (40/343)
$45,000–65,000	–	16.3% (56/343)
$65,000–85,000	–	14% (48/343)
$85,000 or more	–	53.1% (182/343)
Median income		>$85,000
**Education level completed**		
Less than High School (no diploma, certificate, etc.)	–	0.8% (3/369)
High School degree & Equivalent	–	11.9% (44/369)
Some College or University (No degree, certificate)	–	14.9% (55/369)
College, University, Technical degree, Certificate, etc.	–	42.0% (155/369)
Advanced degree, Graduate degree	–	30.4% (112/369)
**Location**		
Urban	23.3% (10/43)^c^	8.4% (31/370)
Rural	48.8% (21/43)	82.2% (304/370)
Semi-rural	16.3% (7/43)	9.5% (35/370)
Unknown	11.6% (5/43)	–
**Children in the household**		
Yes	–	40.2% (149/371)
No	–	59.8% (222/371)

^a^gender assessed in pilot survey by surveyee names.

^b^Question not included in survey.

^c^Rural urban location assessed for pilot survey using google maps imagery of address provided in survey follow-up; “unknown” indicates we never received a reply in follow up.

Nearly all (370/401; 92.3% of respondents) listed eggs as a primary reason for having chickens (Table 2), while 38.4% (154/401) reported keeping chickens as pets, 16% (64/401) reported keeping chickens for meat, and 7.5% (30/401) listed “other.” Of the respondents choosing “other”, the answers varied from breeding/showing (*n* = 3), to bug/pest control (*n* = 15), to composting/fertilizer (*n* = 7). Five respondents had other reasons for keeping poultry. Digging into the reported motivations for keeping poultry, “eggs/meat tastes better than store bought” was the most common motivation (76.1% of respondents; 305/401). “Fun” was the second most common motivation at 53.5% of responses (215/401), while “food independence” (43.4%, 174/401) came in third, and “pest control, like ticks” was fourth (31.9%; 128/401). Four and half percent of the respondents chose other reasons (*n* = 18), including compost/soil, breeding, money, and other, unspecified reasons.

Poultry housing is a key factor in biosecurity, determining whether the birds are likely to come into contact with wild animals and birds or their droppings. The most common housing setups for chickens were a coop with a fixed pen area outside the coop (Table 1; 49.5%; 191/386) or free range outside the coop (46.6%; 180/386). From sampling visits to rural farms, we have noted that many of the fixed penned areas outside coops are not covered and would allow wildlife to enter the area. Only 4.1% of owners kept their chickens entirely indoors (16/386), and 6.7% reported employing a mobile chicken unit (26/386). Eleven percent of owners used a combination of approaches, including both free range and penned areas, or fixed and moveable grazing areas, or a mobile chicken coop in summer with a fixed coop in winter, among others.

Most owners had at least one other species of animal present besides chickens. The most common animals present were dogs and cats, with 67.8% of backyard chicken owners also having dogs (272/401), while 49.1% had cats (197/401). 16.2% of backyard chicken owners also had other species of poultry (65/401), with ducks/geese being the most common additional species (56.9%; 37/65), and turkeys being the second most common additional poultry species (21.5%; 14/65). Besides cats, dogs, and other poultry, horses were the most common animal present alongside chickens (11.2%; 45/401), with goats being the next most common (9.5%; 38/401). Additional species commonly present included cattle (6.2%), sheep (5.5%), pigs (5.5%), and rabbits (4.5%).

Owners fed their chickens a variety of different foods. Commercial feed was the most common food source (93.5%; 375/401), but chickens also frequently received table/food scraps (73.3%; 294/401) and forage (72.3%; 290/401). Given the frequency of free-range housing for birds (40.4%), it is not surprising that forage was a common food option. However, the prevalence of forage as a food source suggests that either some owner with fixed-pen housing believe their chickens have access to forage within those pens (which is possible with a large enough pen) or that they sometimes allow their chickens to free range.

Owners reported a variety of approaches to sick chickens, with “home remedies not specifically natural” being the most common response by a narrow margin (29.2%; 117/401). However, the frequency for “antibiotics/veterinary-prescribed medications,” “natural remedies (herbs, essential oils),” and “put them down” were all in the range of 27.2-29.2%. Among the 6.5% of respondents reporting “other,” 11 reported not treating their birds or letting “nature take its course,” eight reported isolating and monitoring the bird, and seven noted that treatment would depend on various factors.

We asked owners where they got their information on raising chickens, to determine whether official resources were reaching backyard chicken owners in Vermont. Just 26% (98) of owners reported having taken a relevant food microbiology or food safety class or a training that included food safety. Most owners reported getting their information about raising chickens from talking with other chicken owners (67.3%; 270/401), from books (57.9%; 232/401), or from instructional/informational websites or YouTube (57.9%; 232/401). About 21% (84/401) of owners reported getting information from Facebook or social media sources (which vary wildly in quality and accuracy of information), while 16.2% got their information from veterinarians (65/401), and 17.7% reported getting their information from university extension websites or trainings (71/401). UVM Extension's website does not have materials on raising backyard chickens, so owners would probably be accessing materials from other state extension organizations. Finally, 14.7% reported using magazines as a source of information, which is unsurprising, given that magazines such as *Backyard Poultry* are specifically intended for this audience. Just 4.5% (18/401) of respondents chose “other,” with 17 indicating “experience” as a source of information, and one respondent having taken a relevant class. Overall, it seems that CDC/USDA messaging around biosecurity and backyard chickens may not be reaching its target population well.

Perhaps unsurprisingly, given the informal nature of owner education, only a slim majority of owners (60.2%; 228/379) were aware that chickens could carry *S. enterica* (referred to in the survey as “*Salmonella”*) without seeming sick. Over 37% indicated they did not know (37.2%; 141/379), and only 2.6% thought chickens could not carry *Salmonella* without seeming ill (10/379). Knowing which part of the egg was a risk for *S. enterica* was less common; only 41.9% (158/377) indicated *S. enterica* could be either inside or outside the egg, while 12.7% (48/377) thought *Salmonella* was only on the outside, and fully 43% of respondents chose “I don't know” (162/377).

Owners also overall thought backyard chickens were less likely to have issues with *S. enterica* than commercial flocks, with 51.2% (193/377) of respondents indicating that backyard chickens were less likely to have *Salmonella* than commercial flocks. Just 13.8 (52/377) thought backyard chickens were equally likely to have *Salmonella*, while 2.4% (9/377) thought backyard chickens might be more likely to have *Salmonella* than commercial flocks, and nearly 33% replied that they didn't know (123/377). When asked about the risk of *Salmonella* from eggs, results were similar. 40.7% (154/378) of owners thought backyard chicken eggs were less likely to contain *Salmonella* than eggs from the store, 35.4% (134/378) didn't know, 17.7% thought they were equally likely (67/378), and 6.1% (23/378) thought they were more likely to contain *Salmonella* than eggs from the store. Owners were more confident about the safety of eggs from their own flocks, with 46% (174/378) indicating they were safer than eggs from the store, 32.3% (122/378) being unsure, and just 3.2% (12/378) indicating they were less safe.

Unsurprisingly, given this patchy knowledge of chickens being a risk for *S. enterica*, biosecurity practices varied (Table 2). Roughly 75% of owners indicated they habitually washed their hands after handling chickens (304/401) or dirty eggs (300/401), and 68.6% (275/401) reported washing and/or sanitizing their hands after handling any eggs (regardless of dirty/clean appearance). However, fewer than half avoided kissing their birds (47.4%; 190/401) or snuggling their birds (36.4%; 146/401). Only 42.6% of owners (171/401) reported they changed shoes after walking in the chicken area, despite more than 46% of birds having been previously reported to be free range, and just 31.2% of owners reported wearing a mask when cleaning the coop (125/401). Finally, despite 40.2% of respondents reporting having children in the household, just 21.4% (86/401) reported keeping children from snuggling birds, and only 12.2% kept children from interacting with chickens (49/401). However, when asked specifically how often children interacted with their chickens, just 23.5% (88/376) owners indicated that children often or always physically interacted with birds (petting, picking up, etc.), while 42% (158/401) indicated children rarely interact physically with their chickens, and 34.6% of owners indicated children never interact with their chickens (130/401). Anecdotally, when interacting with backyard chicken owners across Vermont, we frequently observed children snuggling or kissing chickens, including in one case a neighbor's child who frequently visited the chickens. Consequently, there is substantial room for improvement in biosecurity habits among families with children.

## Discussion

We found (i) overall, a high proportion of backyard chicken flocks from 2019 to 2021 had *S. enterica*,; (ii) backyard chicken owners were wealthier and more educated than the average Vermonter, but generally lived in rural areas; (iii) participants in the statewide survey had much lower uptake of good biosecurity habits compared to the pilot survey; (iv) despite increased messaging about backyard chicken-associated salmonellosis and good biosecurity measures over the past several years, uptake of biosecurity measures is extremely inconsistent, and rates of unsafe practices such as kissing or cuddling chickens, have increased in Vermont.

### *S. enterica* in Vermont backyard chickens

We found *S. enterica* in 8/42 flocks tested over a 3-year period from June 2019 to December 2021, with 1 positive flock in 2020 (out of 1) and seven positive flocks (out of 13) in 2021. This is a substantially higher rate of *S. enterica* than has been previously reported. McDonagh et al. assessed the prevalence of *S. enterica* in 53 urban backyard chicken flocks in 2016–2017 using a mix of cloacal swabs, dust samples, and fecal samples (5). Just one flock (1.9%) was positive for *S. enterica* (5). A study published in 2019 of backyard poultry flocks in the counties surrounding Seattle, WA, also found *S. enterica* in a single flock (1/34; 2.9%). Finally, a study by the California Animal Health and Food Safety Laboratory System evaluated rates of *S. enterica* in dead chickens submitted for laboratory evaluation from 2012 to 2015 (26). They found *S. enterica* in just 1.6% of birds (37/2,347 birds) over this 3-year period, testing multiple samples from each bird (26). They found *S. enterica* rates did not vary substantially by year, with rates of *S. enterica* ranging from 1.7 to 2.1% of samples from 2012 to 2015 (26). A similar study was performed in Canada by the Animal Health Laboratory of Ontario over a 2-year period, with BYC and small flock owners sending in recently deceased birds for autopsy (27). Two hundred and forty-five chickens were received from a total of 160 farms, and just five farms (3%) had chickens positive for *S. enterica*. Finnish researchers also investigated *S. enterica* prevalence in backyard poultry sent in for necropsy over an 11-year period, and found no birds positive for *S. enterica* *(**28**)*. However, Finnish law requires owners to keep their birds indoors from March to the end of May each year when the wild birds are returning, and this may have reduced *S. enterica* rates in backyard chickens (28). Additionally, it is possible that Finnish hatcheries have eliminated *S. enterica* from their breeder flocks, which would substantially reduce prevalence in backyard flocks, since Finland prohibits the importation of poultry (28). The only study which found similar rates of *S. enterica* in backyard chickens was performed in Australia and found *Salmonella* in 10.4% of flocks (4/30) (25). Consequently, our study presents a massive increase in *S. enterica* over previous studies in North America.

A potential reason for this sudden increase in *S. enterica* in backyard flocks in 2021 is the outbreak of salmonellosis from *S. enterica* serovar Typhimurium in songbirds, specifically in Pine Siskins, a species whose range includes Vermont (29–31). This outbreak was reported in April 2021, and led to 29 illnesses across 12 states in the United States, including New Hampshire (32). While no illnesses were reported in Vermont, the Vermont State Department of Health had seconded all their personnel to COVID-19 response; consequently, salmonellosis cases were not investigated and therefore not reported (33). Intriguingly, 4/7 positive flocks in 2021 tested positive during the songbird outbreak period. Further research is needed to determine whether these *S. enterica* isolates are similar to the outbreak strain.

### Survey data

More than 80% (83.7%; 36/43) of respondents to the pilot survey were female, compared with 66.7% of the statewide survey respondents (Table 4). Just over half (55.3%; 21/38) of the pilot survey respondents with available address information were living in rural areas, as assessed by Google Maps imagery. In contrast, 82.2% of respondents in the statewide survey indicated they lived in rural areas. We did not ask about education or income in the pilot survey. The most recent census data (2021) reported 260,029 households in Vermont, meaning our statewide survey represents 0.67% of Vermont households (22). Highly educated Vermont residents were over-represented in our survey; 38% of Vermonters overall have a Bachelor's degree or higher in Vermont, with 52.6% of residents aged 25–64 having at least a technical degree or certificate beyond high school (34), compared with 72% of our survey population with at least a technical degree or certificate beyond high school (22). In tandem, the majority (67.1%) of our owner pool had a higher income than Vermont's median salary of $61,973 (22).

Overall, the data suggest that backyard chicken owners in Vermont are more highly educated and earn a higher income than the average Vermonter. This is similar to the findings of McDonagh et al. in their survey of backyard chicken owners in Massachusetts. In their sample, 79.6% (39/49) chicken owners had a household income of $100,001–>200,000 per year, compared to the 2016–2020 median income for Boston of $76,298, and an equal number had a graduate degree. Kauber et al.'s study in Seattle did not ask about income, but also found that 48% (24/50) of their survey respondents had a graduate degree (12). In a 2014 survey of backyard chicken owners across the United States (though 61% of respondents were from California), the majority of respondents were also female and highly educated, with 67% of respondents having completed a 4 year degree or higher (35). Additionally, 41.2% of respondents had incomes of >$100,000 per year (35). This was perhaps influenced by the preponderance of responses from California, but very few respondents, even in rural areas, indicated they kept birds for income (35). Similarly, a recent nationwide survey of backyard poultry owners in France found backyard poultry owners were most commonly middle-aged and nearly 30% were in senior management, suggesting a comfortable income (36). Consequently, efforts to educate backyard chicken owners, should take advantage of their target audience's education to create nuanced materials that reflect the level of uncertainty around the actual risk of backyard poultry to the owners.

Despite the high levels of education, populations surveyed do not fully understand the risks associated with backyard chickens. Most (83.7%; 36/43) of owners in the pilot study thought chickens could have *Salmonella* without seeming ill, and 60.2% (228/379) of respondents to the statewide survey answered “yes” to the same question. In the pilot survey, only 51% of respondents knew that *Salmonella* could be inside an egg, while only 41.9% of the statewide survey respondents were aware of this. In the pilot survey, 30% of respondents chose the “unsure” response to this question, while 43% of the statewide survey respondents chose “I don't know” in response to this question. When asked about the relative likelihood of *S. enterica* in backyard flocks vs. commercial flocks, 65.1% of owners in the pilot survey thought backyard chickens were less likely to have *Salmonella* and 25.6% thought there was no difference in likelihood. In contrast, in the statewide survey, only 51.2% thought backyard chickens were less likely, and 13.8% thought they were equally likely to have *Salmonella*. However, the pilot survey did not have an “I don't know” option for this question, which may have influenced the results, as for the statewide survey, 32.6% of respondents chose this option.

Unsurprisingly, owners of backyard chickens inconsistently employ risk-mitigation measures. Seattle, Boston, and Canadian (Ontario) owners washed hands after handling chickens or ducks 98, 65.3, and 94% of the time, respectively, (10, 12, 37) while 84.1% of chicken owners in our pilot study and 75.8% of owners in our statewide study reported washing their hands after handling their chickens (Table 5). The rates of handwashing after handling chickens were likely higher in our pilot study than our statewide study due to the higher percentage of urban owners in our pilot study or due to the higher engagement/interest in biosecurity required of owners in the pilot study. However, this does not explain the lower rates of handwashing in Boston vs. Seattle and Ontario, Canada. Similarly, 86% of Seattle owners reported washing their hands after handling raw eggs, while 65.3% of Boston owners (10), 60.5% of our pilot study respondents, and 68.6% of our statewide survey respondents washed their hands after handling eggs. The reason for this disparity between Seattle and the Northeast is unclear. In contrast, masking while cleaning the coop was adopted at similar rates in Seattle and Vermont; 28% (13/47) of Seattle BYC owners reported wearing a mask to clean their coops (12), compared with 18.6% of owners in the pilot survey and 31.2% of owners in the statewide study. The small increase in masking from our pilot study to our statewide study may be due to the increased accessibility and use of masks overall during the COVID-19 pandemic.

**Table 5:** Comparison of our survey data with previous studies.

**Biosecurity practices**	**Kauber^a^**	**McDonagh^b^**	**Brochu^c^**	**This study (pilot)**	**This study (statewide)**
	**% (Number/total)**	**% (Number/total)**	**% (Number/total**	**% (Number/total)**	**% (Number/total)**
Hug/kiss/snuggle birds or eat/drink/smoke, touch mouth around birds	22% (11/50)	–^d^	–	–	–
Avoid hugging/kissing/snuggling birds or eating/drinking/smoking, touching face around birds	78% (39/50)	–	–	–	–
Kiss birds	–	10% (3/30)	–	*9.3% (4/43)^*e*^*	*52.6% (211/401)*
Avoid kissing birds	–	90% (27/30)	–	90.7% (39/43)	47.4% (190/401)
Hug/snuggle birds	–	40% (12/30)	–	*41.9% (18/43)*	*63.6% (255/401)*
Avoid hugging/snuggling birds	–	60% (18/30)	–	58.1% (25/43)	36.4% (146/401)
Children hug/snuggle birds	–	47.8% (11/23)	–	–	23.5% (88/376)
Keep children from hugging/snuggling birds	–	52.2% (12/23)	–	37.2% (16/43)	21.4% (86/401)
Keep children from interacting with birds	42.6% (20/47)	0% (0/33)	–	9.3% (4/43)	12.2% (49/401)
Wash hands after handling eggs	86% (43/50)	65.3% (32/50	–	60.5% (26/43)	68.6% (275/401)
Wash hands after handling chickens	98% (49/50)	65.3% (32/50)	93.8% (137/146)	84.1% (37/43)	75.8% (304/401)
Wear a mask when cleaning coop	27.7% (13/47)	–	–	18.6% (8/43)	31.2% (125/401)
Change shoes after walking in chicken area	–	–	38.5% (47/122)	51.2% (22/43)	42.6% (171/401)

^a^Kauber et al. (12).

^b^McDonagh et al. (10).

^c^Brochu et al. (37).

^d^“–” indicates data not obtained in that survey.

^e^Numbers in italics are inferred for the pilot and statewide survey and are the number of respondents who did not indicate they followed that biosecurity habit.

Risk factors for acquiring *S. enterica* from live poultry include picking up birds, kissing birds, and snuggling birds (4). In Seattle, 22% of owners admitted to snuggling, kissing, eating/drinking, or touching their face around their adult chickens and 26% admitted to the same practices around chicks (Table 5) (12). Additionally, 24% allowed poultry to live in their houses or to have access to patios or kitchens (12). In Boston, the authors found that nearly all (96.5%) of owners picked up their birds, while only 41.4% hugged them and just 10.3% kissed their birds (10). Additionally, 68.9% of children picked up their chickens, 37.9% of children hugged chickens and 17.2% of children kissed chickens (10).

In our pilot study, we found numbers similar to McDonagh's for snuggling and kissing; 58.1% avoided snuggling their birds, and 90.7% avoided kissing their birds, suggesting that 41.9% may have snuggled their birds, and 9.3% may have kissed their birds. However, in our statewide survey, we found substantially higher rates of probable snuggling, with only 36.4% of owners indicating they avoided snuggling their birds, and only 47.4% of owners indicating they avoided kissing their birds. We explored whether the decrease in good biosecurity habits was associated with a higher number of rural respondents, but we found that overall, rural participants in the statewide study were slightly *more* likely to adopt good biosecurity habits, compared to urban participants (though this could be due to having only 31 urban participants in the statewide survey; Supplementary Table S3). Finally, 40% of our sample had children in their household, and 23.5% of owners had children often or always petting or picking up their birds (χ^2^ = *p* < 0.001). This is relevant, as children are considered more likely to develop salmonellosis, and indeed, the Vermont Department of Health investigations on live-poultry associated salmonellosis found that 30.2% of patients were under 10 years old (4, 21).

Significantly, the implied rates of bad biosecurity habits are overall similar to the exposure characteristics found in a compilation of characteristics connected with *Salmonella* cases from live-poultry exposure; 59% of live poultry-associated salmonellosis patients had held/snuggled their birds, and 13% had kissed their birds (4). This indicates that outreach efforts since 2013 have affected minimal to no change in poultry owners' interactions with their birds, which is supported by the low reach of university extension websites in Vermont (just 17.7%) to backyard chicken owners. We did not specifically ask about government websites, such as the CDC's Healthy Pets, Healthy People web page on backyard poultry (32), so it is unclear how much reach these have had, though based on the survey responses, governmental websites are unlikely to be reaching more than 60% of the backyard chicken owners in Vermont.

The CDC recently ran a series of focus groups with backyard poultry owners (38). The key finding was that despite the fact that all participants were aware that kissing chickens was risky and handwashing was important, participants were unwilling to reduce physical contact with poultry (38). This correlates with the relatively low percentage of owners avoiding kissing and snuggling their birds in our study, and to a lesser extent, in the Boston study (10). One reason for this was that the CDC focus group participants didn't believe they were at risk for salmonellosis, preferring to view themselves as “responsible owners,” and did not view salmonellosis as a genuine threat (38). Additionally, participants indicated that risk-based messaging was not persuasive (as is also obvious from studying backyard chicken social media pages after a CDC outbreak report). This may indicate that public health entities should move away from a risk-based focus to a more positive messaging style that encourages best practices. Indeed, when the CDC asked poultry owners about the style of messaging they preferred, participants preferred “visually appealing and eye-catching images [and] layout,” ideally with pictures of baby chicks and disliked negative messaging about the number of live poultry-associated outbreaks (38). Ultimately, it is still important for flock owners to know that there are risks, so perhaps providing hard numbers on the frequency of *S. enterica* in backyard chickens and the most beneficial and easy-to-implement biosecurity strategies to reduce risk would strike the balance of informing and encouraging without employing “scare tactics.” Further, tweaking federal messaging to increase its relevancy for the state or local context, and increasing the use of social media outreach (for instance, on Instagram) could increase the potency and reach of biosecurity messaging. An example of the way forward is potentially the U.S. National Park Service's Instagram page (https://www.instagram.com/nationalparkservice/). Combining humor, pop culture references, and facts (e.g., the danger of approaching bison and other wildlife or facts on wildlife), it has attracted a substantial following (4.1 million followers as of June 2022) and has high engagement with its posts.

In addition to determining how to reformulate biosecurity messaging to be more persuasive, the biosecurity risk of bird feeders adjacent to domestic poultry should be included in future messaging. While the uptick in Salmonellosis in Vermont backyard birds may or may not be related to the songbird outbreak of 2021 (39), a recent study in Georgia found that wild birds frequent chicken coops where there is accessible chicken feed (40). Northern Cardinals, a species commonly affected by *S. enterica* outbreaks, spent the most time around chicken coops, which demonstrates a strong potential for spillover infections into backyard poultry (40, 41). Further, a study in Canada found that 10% of all European Starlings and House Sparrows collected near broiler houses were positive for *S. enterica* (42). Consequently, biosecurity messaging should include the importance of keeping poultry feeding stations inaccessible to wild birds (i.e., inside the coop) and not having bird feeders in the same yards as domestic poultry. This could be framed as both protecting the wild birds from acquiring *S. enterica* from the poultry, as well as protecting the poultry from the wild birds, without passing undue blame on either species.

## Conclusions and future directions

Despite several years of messaging campaigns from state and national health organizations, little has changed in the habits and perceptions of backyard chicken owners. This indicates an acute need for more effective communication surrounding biosecurity practices. Meanwhile, the *S. enterica* rate in backyard chickens fluctuates wildly, for reasons that are still unclear. Key problems to solve in future include (i) increasing the reach of information around biosecurity best practices and (ii) increasing the effectiveness of this messaging. Additionally, backyard chicken owners need clearer information on the risks associated with close contact with domestic poultry, so they can make informed decisions. This requires ongoing investigation on *S. enterica* rates in different contexts to determine which factors (e.g., temperature, season, housing, feed, wildlife exposure, etc.) increase the likelihood of *S. enterica* in backyard poultry. Future directions for this project include completing sequencing of the *S. enterica* isolates from this research to determine whether they are from serovars which commonly cause human illness and working with communications professionals to develop and test messaging relevant to Vermonters/the Northeast in both rural and urban contexts.

## Data availability statement

All datasets presented in this study are included in the article/Supplementary material.

## Ethics statement

The studies involving human participants were reviewed and approved by University of Vermont Institutional Review Board. Written informed consent for participation was not required for this study in accordance with national legislation and the institutional requirements. The animal study was reviewed and approved by University of Vermont Institutional Care and Animal Use Committee. Written informed consent for participation was not obtained from the owners because Surveyees were asked at the end if they wished to have their birds sampled, with a brief explanation of the cloacal swab process. Owners who indicated they were interested were contacted to set up a sampling time.

## Author contributions

MD collected and processed the majority of the survey and biological data. KL assisted with sample processing, data management, and edits during manuscript preparation and publishing. KH assisted with data management and arranging samplings. AE conceived the project, supervised and trained MD, KL, and KH, analyzed the data, and wrote the manuscript. All authors contributed to the article and approved the submitted version.

## Funding

This research was funded by the Vermont Agricultural Experiment Station-Hatch funds startup funds (2019–2020) and by Award No.VT-H02812MS (2021-2024) as part of Multistate Project S1077.

## Conflict of interest

The authors declare that the research was conducted in the absence of any commercial or financial relationships that could be construed as a potential conflict of interest.

## Publisher's note

All claims expressed in this article are solely those of the authors and do not necessarily represent those of their affiliated organizations, or those of the publisher, the editors and the reviewers. Any product that may be evaluated in this article, or claim that may be made by its manufacturer, is not guaranteed or endorsed by the publisher.
