# Supplementary material for: Salmonella enterica frequency in backyard chickens in Vermont and biosecurity knowledge and practices of owners
Source: Front Vet Sci. 2022 Sep 22;9:979548. doi: 10.3389/fvets.2022.979548 (PMC9536149; doi:10.3389/fvets.2022.979548)
Supplement: Supplementary file 2 [file Table_2.docx]

2022 Vermont Back Yard Chicken Survey- univariate results.

Towns analysis provided in associated excel doc.

Do you currently have a backyard ﬂock of chickens or have you had a flock in the last year?

|  | Frequency | Percent |
| --- | --- | --- |
| Yes | 401 | 23.2 |
| No | 1329 | 76.8 |
| Total | 1730 | 100.0 |

About how many chickens do you have now, or have you had on average over the past year?

| Mean | 19.5 |
| --- | --- |
| Median | 10.0 |
| Mode | 6.00 |

Do you have chickens for: (Select any that apply)

| N = 401 | Frequency | Percent |
| --- | --- | --- |
| Eggs | 370 | 92.3 |
| Meat | 64 | 16.0 |
| Pets | 154 | 38.4 |
| Another Reason | 30 | 7.5 |

Other reasons for having chickens

| N = 30 | Frequency | Percent |
| --- | --- | --- |
| Breeding/Showing | 3 | 10.0 |
| Bug/Pest Control | 15 | 50.0 |
| Composting/Fertilizer | 7 | 23.3 |
| Other | 5 | 16.7 |

What are your primary reasons for keeping chickens? (Select any that apply)

| N = 401 | Frequency | Percent |
| --- | --- | --- |
| Eggs/Meat tastes better than store bought | 305 | 76.1 |
| Pest control, like ticks | 128 | 31.9 |
| Food independence | 174 | 43.4 |
| Fun | 215 | 53.6 |
| Another reason (9 Compost/Soil, 4 Breeding, 3 Other, 2 Money) | 18 | 4.5 |

How are your chickens primarily housed? (Besides their coop)

|  | Frequency | Percent |
| --- | --- | --- |
| Coop with free range beyond a penned area | 156 | 40.4 |
| Coop with a fixed penned in area | 156 | 40.4 |
| Indoor housing only (inside a building, coop, etc.) | 8 | 2.1 |
| Coop with a movable penned in area (mobile chicken unit, etc.) | 22 | 5.7 |
| Some other housing option (All answers were combinations) | 44 | 11.4 |
| Total | 386 | 100.0 |

What are your chicken's food source(s)? (Select any that apply)

| N = 401 | Frequency | Percent |
| --- | --- | --- |
| Forage (natural ground area) | 290 | 72.3 |
| Commercial feed (purchased from a store) | 375 | 93.5 |
| Table scraps/food scraps | 294 | 73.3 |
| Another food source (14 Home Grown food, 6 Bird seed and other foods not commercial feed) | 20 | 5.0 |

Do you have any other animals? (Select any that apply)

| N = 401 | Frequency | Percent |
| --- | --- | --- |
| Dogs | 272 | 67.8 |
| Cats | 197 | 49.1 |
| Horses | 45 | 11.2 |
| Cattle | 25 | 6.2 |
| Sheep | 22 | 5.5 |
| Goats | 38 | 9.5 |
| Pigs | 22 | 5.5 |
| Other Poultry | 65 | 16.2 |
| Other Animals | 46 | 11.5 |

Other Poultry

| N = 65 | Frequency | Percent |
| --- | --- | --- |
| Ducks/Geese | 37 | 56.9 |
| Turkeys | 14 | 21.5 |
| Guinea Fowl | 10 | 15.4 |
| Quail | 2 | 3.1 |
| Other Birds | 2 | 3.1 |

Other Animals

| N = 46 | Frequency | Percent |
| --- | --- | --- |
| Rabbits | 18 | 39.1 |
| Lizard/Tortoise/Fish/Crab | 10 | 21.7 |
| “Domestic” Birds | 7 | 15.2 |
| Alpaca/Emu/Donkey/Mule | 5 | 10.9 |
| Bees | 2 | 4.3 |
| Guinea Pig/Hedgehog | 2 | 4.3 |
| Other Animals | 2 | 4.3 |

How do you treat your chickens if they are sick? (Select any that apply)

| N = 401 | Frequency | Percent |
| --- | --- | --- |
| Antibiotics/veterinary-prescribed medicines | 109 | 27.2 |
| Natural remedies (herbs, essential oils) | 113 | 28.2 |
| Home remedies not speciﬁcally "natural" | 117 | 29.2 |
| Put them down | 113 | 28.2 |
| Some other treatment (11 Do not treat/let nature take its course, 8 isolate/monitor, 7 treatment is dependent.) | 26 | 6.5 |

How do you typically process eggs from your backyard chickens?

|  | Frequency | Percent |
| --- | --- | --- |
| Wash and Refrigerate | 153 | 42.1 |
| Don't wash and do Refrigerate | 95 | 26.2 |
| Don't wash and do not Refrigerate | 99 | 27.3 |
| Wash and do not Refrigerate | 16 | 4.4 |
| Total | 363 | 100.0 |

What habits do you follow when handling your chickens or their eggs? (Select any that apply)

| N = 401 | Frequency | Percent |
| --- | --- | --- |
| Wash hands after handling chickens | 304 | 75.8% |
| Wash hands after handling dirty eggs | 300 | 74.8% |
| Wash hands after handling eggs (and sanitizer) | 275 | 68.6% |
| Avoid kissing birds | 190 | 47.4% |
| Change shoes after walking in chicken area (and clothes) | 171 | 42.6% |
| Avoid snuggling birds (and touching) | 146 | 36.4% |
| Wear a mask when cleaning the chicken coop (and goggles) | 125 | 31.2% |
| Keep children from snuggling birds | 86 | 21.4% |
| Keep children from interacting with chickens | 49 | 12.2% |
| Wear gloves when handling chickens/eggs | 33 | 8.2% |
| Something else (4 avoid cross-contamination with other flocks, 2 wash eggs, 2 keep very clean coop, 4 other) | 12 | 3.0% |

How often do children physically interact (petting, picking up, etc.) with your chickens? (Besides getting eggs)

|  | Frequency | Percent |
| --- | --- | --- |
| Never | 130 | 34.6 |
| Rarely | 158 | 42.0 |
| Often | 66 | 17.6 |
| Always | 22 | 5.9 |
| Total | 376 | 100.0 |

Now we have a few questions about your opinions of salmonella and chickens. Please provide your answers for the following health statements.

In your opinion, can chickens carry Salmonella while appearing healthy?

|  | Frequency | Percent | |
| --- | --- | --- | --- |
| Yes | 228 | 60.2 |  |
| No | 10 | 2.6 |  |
| I don't know | 141 | 37.2 |  |
| Total | 379 | 100.0 |  |

Can an egg have Salmonella on the inside, outside or both?

|  | Frequency | Percent |
| --- | --- | --- |
| Inside | 9 | 2.4 |
| Both inside or outside | 158 | 41.9 |
| Outside | 48 | 12.7 |
| I don't know | 162 | 43.0 |
| Total | 377 | 100.0 |

In your opinion, are backyard ﬂocks more or less likely to have Salmonella than commercial ﬂocks?

|  | Frequency | Percent |
| --- | --- | --- |
| More Likely | 9 | 2.4 |
| Equally Likely | 52 | 13.8 |
| Less Likely | 193 | 51.2 |
| I don't know | 123 | 32.6 |
| Total | 377 | 100.0 |

In your opinion, are eggs from backyard ﬂocks more or less likely to contain Salmonella than eggs from the store?

|  | Frequency | Percent |
| --- | --- | --- |
| More likely to contain salmonella than eggs from the store | 23 | 6.1 |
| Equally likely to contain salmonella | 67 | 17.7 |
| Less likely to contain salmonella than eggs from the store | 154 | 40.7 |
| I don't know | 134 | 35.4 |
| Total | 378 | 100.0 |

In your opinion, are eggs from your chickens more or less likely to contain Salmonella than eggs from the store?

|  | Frequency | Percent |
| --- | --- | --- |
| More likely to contain salmonella than eggs from the store | 12 | 3.2 |
| Equally likely to contain salmonella | 70 | 18.5 |
| Less likely to contain salmonella than eggs from the store | 174 | 46.0 |
| I don't know | 122 | 32.3 |
| Total | 378 | 100.0 |

Have you ever taken a food safety or food microbiology class or taken part in a training that included information on food safety?

|  | Frequency | Percent |
| --- | --- | --- |
| I have taken a relevant class | 58 | 15.4 |
| I have participated in a relevant training | 40 | 10.6 |
| I have not taken a relevant class or training | 279 | 74.0 |
| Total | 377 | 100.0 |

How do you get information about raising chickens?

| N = 401 | Frequency | Percent |
| --- | --- | --- |
| Talking with others who raise chickens | 270 | 67.3 |
| Books about raising chickens | 232 | 57.9 |
| Instructional/informational Websites, Youtube, etc. | 232 | 57.9 |
| Facebook or other social media sources | 84 | 20.9 |
| University Extension Education materials, website, trainings | 71 | 17.7 |
| Veterinarians | 65 | 16.2 |
| Magazines | 59 | 14.7 |
| Some other source (17 experience, 1 took a class.) | 18 | 4.5 |

We just have a few demographic questions to help us better understand who in Vermont is raising backyard chickens.

Do you have people in your household who are under 18 years of age?

|  | Frequency | Percent |
| --- | --- | --- |
| Yes | 149 | 40.2 |
| No | 222 | 59.8 |
| Total | 371 | 100.0 |

Select the option that best describes the neighborhood where you have chickens.

|  | Frequency | Valid Percent |
| --- | --- | --- |
| Rural countryside | 304 | 82.2 |
| Suburban village or housing development | 35 | 9.5 |
| Urban city or town | 31 | 8.4 |
| Total | 370 | 100.0 |

Please write in your gender identification.

|  | Frequency | Percent |
| --- | --- | --- |
| Male | 105 | 32.4 |
| Female | 216 | 66.7 |
| Trans/non-binary | 3 | 0.9 |
| Total | 324 | 100.0 |

Please select the category that includes your age.

|  | Frequency | Percent |
| --- | --- | --- |
| 18-24 | 1 | .3 |
| 25-34 | 21 | 6.0 |
| 35-44 | 60 | 17.1 |
| 45-54 | 82 | 23.4 |
| 55-64 | 93 | 26.6 |
| 65 or over | 93 | 26.6 |
| Total | 350 | 100.0 |

Please select the category that best represents your total household income after taxes in 2020.

|  | Frequency | Percent |
| --- | --- | --- |
| $25,000 or less | 17 | 5.0 |
| $25,000-$45,000 | 40 | 11.7 |
| $45,000- $65,000 | 56 | 16.3 |
| $65,000-$85,000 | 48 | 14.0 |
| $85,000 or more | 182 | 53.1 |
| Total | 343 | 100.0 |

What is the highest level of education you’ve completed?

|  | Frequency | Percent |
| --- | --- | --- |
| Less than High School (no diploma, certificate, etc.) | 3 | .8 |
| High School degree & Equivalent | 44 | 11.9 |
| Some College or University (No degree, certificate) | 55 | 14.9 |
| College, University, Technical degree, Certificate, etc. | 155 | 42.0 |
| Advanced degree, Graduate degree | 112 | 30.4 |
| Total | 369 | 100.0 |

Finally, have you had diarrheal symptoms lasting more than a day in the last year, or since you began raising chickens?

|  | Frequency | Percent |
| --- | --- | --- |
| Yes | 23 | 6.3 |
| No | 345 | 93.8 |
| Total | 368 | 100.0 |

Crosstabulations:

| Chi sq. = .019 (High Correlation) | | How are your chickens primarily housed? (Besides their coop) - Selected Choice | | | | | Total |
| --- | --- | --- | --- | --- | --- | --- | --- |
|  |  | Coop with free range beyond a penned area | Coop with a fixed penned in area | Indoor housing only (inside a building, coop, etc.) | Coop with a movable penned in area (mobile chicken unit, etc.) | Some other housing option (Please describe) |  |
| Select the option that best describes the neighborhood where you have chickens. | Rural countryside | 85.0% | 78.4% | 37.5% | 86.4% | 90.5% | 82.0% |
|  | Suburban village or housing development | 8.2% | 10.1% | 37.5% | 13.6% | 4.8% | 9.5% |
|  | Urban city or town | 6.8% | 11.5% | 25.0% |  | 4.8% | 8.4% |
| Total | | 100.0% | 100.0% | 100.0% | 100.0% | 100.0% | 100.0% |

| Chi sq. = <.001 (High Correlation) | | Do you have people in your household who are under 18 years of age? | | Total |
| --- | --- | --- | --- | --- |
|  |  | Yes | No |  |
| How often do children physically interact (petting, picking up, etc.) with your chickens? (Besides getting eggs) | Never | 14.1% | 47.9% | 34.6% |
|  | Rarely | 45.1% | 41.6% | 42.9% |
|  | Often | 30.3% | 8.7% | 17.2% |
|  | Always | 10.6% | 1.8% | 5.3% |
| Total | | 100.0% | 100.0% | 100.0% |
