# Supplementary material for: Salmonella enterica frequency in backyard chickens in Vermont and biosecurity knowledge and practices of owners
Source: Front Vet Sci. 2022 Sep 22;9:979548. doi: 10.3389/fvets.2022.979548 (PMC9536149; doi:10.3389/fvets.2022.979548)
Supplement: Supplementary file 4 [file Data_Sheet_1.pdf]

Poultry-Project

Survey Flow

Standard: Default Question Block (30 Questions)

Page Break

Start of Block: Default Question Block

Q35

Research Information Sheet

Title of Study: Assessing Factors that Lead to *Salmonella* in VT Backyard Flocks of Chickens
Principal Investigator (PI): Dr. Andrea Etter
Funder: VT Agricultural Experiment Station

Introduction You are being invited to take part in this research study because you live in VT and are planning on purchasing hatchling chicks for a backyard flock this spring or already have backyard chickens. This study is being conducted by Andrea Etter at the University of Vermont.

Purpose The purpose of this study is to collect information on husbandry practices and flock features in order to determine what factors, if any, affect the prevalence of *Salmonella* in backyard flocks of chickens in VT and how common *Salmonella* is in hatchling chicks and backyard chickens.

Study Procedures If you take part in the study, you will be asked to fill out a one-time simple online survey. This should take no more than ten minutes of your time. We will need your name and contact information in order to conduct a one-time visit to your farm/home to perform cloacal swabs on your hatchlings and backyard chickens.

Benefits As a participant in this research study, there is unlikely to be any direct benefit for you; however, information from this study may benefit other people now or in the future.

Risks We will do our best to protect the information we collect from you during this study. We will not collect any information that will identify you to further protect your confidentiality and avoid any potential risk for an accidental breach of confidentiality.

Costs There will be no costs to you for participation in this research study.

Compensation You will not be paid for taking part in this study. We thank you for your time!

Confidentiality All information collected about you during the course of this study will be stored with a code name or number so that we are able to match you to your answers. We will keep your information on a secure computer to protect it. Data will be published without any personal identifiers.

Voluntary Participation/Withdrawal Taking part in this study is voluntary. You are free to not answer any questions or withdraw at any time. You may choose not to take part in this study, or if you decide to take part, you can change your mind later and withdraw from the study. If you withdraw without completing the survey, your data will be excluded from the study.

Questions If you have any questions about this study now or in the future, you may contact me (**Andrea Etter)** at the following phone number: **(802)-656-0541** or via email at **andrea.etter@uvm.edu** with the subject line "hatchling chick project"or "backyard chicken project"**.** If you have questions or concerns about your rights as a research participant, then you may contact the Director of the Research Protections Office at (802) 656-5040.

Participation Your participation is voluntary, and you may refuse to participate without penalty or discrimination at any time. Please print this information sheet for your records before continuing.

Q01 Do you have a backyard flock of chickens?

○ Yes (1)

○ No (2)

Skip To: End of Survey If Q01 = No

Q02 What ages of chickens do you have? Select all that apply.

☐ Adult chickens (1)

☐ Hatchlings (2)

Skip To: Q04 If Q02 != Hatchlings

Q03 What hatchery/supplier did you get your chicks from?

____

____

____

____

____

Q04 Do you sell eggs?

○ Yes (1)

○ No (2)

Skip To: Q05 If Q04 = Yes

Q05 Who do you sell eggs to?

- ☐ A business (store/restaurant/grocery) (4)
- ☐ Friends (5)
- ☐ Family (6)
- ☐ At a farmer's market (8)
- ☐ In a road side stand (9)

Skip To: End of Survey If Q05 = A business (store/restaurant/grocery)

Skip To: End of Survey If Q05 = At a farmer's market

Q06 Do you have more than 30 chickens?

- ○ Yes (1)
- ○ No (2)

Q07 How many chickens do you have?

____

Q08 Where did you get your chickens from?

- ○ Commercial hatchery (1)
- ○ Friend/acquaintance (2)
- ○ Hatched from hens in own flock (3)
- ○ Other (4) ____

*

Q09 What breed(s) are your chickens? Please include number of chickens from each breed in the text boxes

- ☐ Barred Rock (1) ________________
- ☐ Plymouth Rock (2) ________________
- ☐ Ameraucauna (3) ________________
- ☐ Rhode Island White (4) ________________
- ☐ Rhode Island Red (5) ________________
- ☐ Banty (6) ________________
- ☐ Cochin (7) ________________
- ☐ Orpinton (any color) (8) ________________
- ☐ Leghorn (9) ________________
- ☐ Wyandotte (10) ________________
- ☐ Australorp (11) ________________
- ☐ Sussex (12) ________________
- ☐ Brahma (13) ________________
- ☐ Golden Comet/other hybrid (specify) (14) ________________
- ☐ Pekin (15) ________________
- ☐ Other breed (specify) (16) ________________

Q10 How are your chickens housed?

- ○ Free range (1)
- ○ Penned (fixed area) (2)
- ○ Indoor housing only (3)
- ○ Penned in moving area (e.g., mobile chicken unit) (4)
- ○ Other (specify) (5) ____

Q11 What are your chicken's primary food source(s)? check all that apply

- ▢ Forage (1)
- ▢ Commercial feed (2)
- ▢ Table scraps/food scraps (3)
- ▢ Other (specify) (4) ____

Q12 Do you have other animals?

- ▢ horses (1)
- ▢ cattle (2)
- ▢ sheep (3)
- ▢ goats (4)
- ▢ pigs (5)
- ▢ cats (6)
- ▢ dogs (7)
- ▢ other poultry (specify) (8) ____
- ▢ Other animals (specify) (9) ____
- ▢ no other animals (10)

Q13 Do your chickens potentially have contact with wildlife?

- ○ Definitely yes (1)
- ○ Probably yes (2)
- ○ Might or might not (3)
- ○ Probably not (4)
- ○ Definitely not (5)

Q14 How do you treat your chickens if they are sick?

- ○ Antibiotics/veterinary-prescribed medicines (1)
- ○ natural remedies (herbs, essential oils) (2)
- ○ Home remedies not specifically "natural" (3)
- ○ Put bird down (4)
- ○ Other (5) ____

Q15 How do you typically handle eggs from your backyard chickens?

- ○ Wash, refrigerate (1)
- ○ Don't wash, but refrigerate (2)
- ○ Wash, don't refrigerate (3)
- ○ Don't wash, don't refrigerate (4)

Skip To: Q16 If Q15 = Don't wash, but refrigerate

Skip To: Q16 If Q15 = Wash, refrigerate

Skip To: Q17 If Q15 = Wash, don't refrigerate

Q16 Describe your rationale for not washing or refrigerating the eggs from your backyard flock

____

Q17 What habits do you follow when handling your chickens or their eggs? (check all that apply)

- ☐ Wash your hands after handling chickens (1)
- ☐ Wash your hands after handling eggs (2)
- ☐ Wash hands after handling eggs ONLY if eggs were dirty (3)
- ☐ Change shoes after walking around chicken area (4)
- ☐ Wear latex gloves (5)
- ☐ Wear a mask when cleaning the chicken coop (6)
- ☐ Avoid kissing birds (7)
- ☐ Avoid snuggling birds (8)
- ☐ Keep children from snuggling birds (9)
- ☐ Keep children from interacting with chickens (10)
- ☐ Other (describe) (11) ____

Q18 What are your primary reasons for keeping chickens? (drag into order)
______ Homegrown eggs are healthier (1)
______ Homegrown eggs are tastier (2)
______ Homegrown eggs are more sustainable (3)
______ Good learning experience for children (4)
______ Pets/companionship (5)
______ Other (explain) (6)

Q19 Now, we'd like to ask a few questions about your knowledge of *Salmonella* and chickens

---

Q20 Chickens can carry *Salmonella* while appearing healthy

- Yes (1)
- Maybe (2)
- No (3)

Q21 In your opinion, are backyard flocks more or less likely to have *Salmonella* than commercial flocks?

- More (1)
- Less (2)
- No difference (3)

Q22 In your opinion, are chickens purchased from a commercial hatchery more or less likely to be infected with *Salmonella*?

- More (1)
- Less (2)
- No difference (3)

Q23 In your opinion, are eggs from backyard flocks more or less likely to contain *Salmonella* than eggs from the store?

- More (1)
- Less (2)
- No difference (3)

Q24 In your opinion, are eggs from ***your*** chickens more or less likely to contain *Salmonella* than eggs from the store?

- ○ More (1)
- ○ Less (2)
- ○ No difference (3)

Q25 In your opinion, is it safer to eat raw egg products (cookie dough, egg nog, etc) made from **your** backyard flock's eggs than from store-bought eggs?

- ○ Yes (1)
- ○ No (2)
- ○ Maybe (3)

Q26 A chicken infected with *Salmonella* can transmit *Salmonella* directly into the inside of the eggs she lays

- ○ True (1)
- ○ False (2)
- ○ Unsure (3)

Q27 Have you had diarrheal symptoms in the last year or since you began raising chickens (whichever is more recent)?

- ◯ Yes (1)
- ◯ I don't remember (2)
- ◯ No (3)

Q28 Are you willing to have Dr. Etter and a student perform a one time cloacal swab on your chickens?
(This is a non-invasive procedure which simply involves inserting a swab briefly into the bird's cloaca (anus) to collect a fecal sample. Dr. Etter has experience with chickens and will ensure your birds are not harmed.)

- ◯ Yes (1)
- ◯ Maybe--want more information (2)
- ◯ No (4)

Q29 Please enter your name and contact information so that we can set up an on-farm visit to collect samples from your chickens

- ◯ Name (1) ____
- ◯ email address (2) ____
- ◯ phone number where you can be reached (3) ____
- ◯ City (4) ____
- ◯ State (5) ____

End of Block: Default Question Block
