# Supplementary material for: Salmonella enterica frequency in backyard chickens in Vermont and biosecurity knowledge and practices of owners
Source: Front Vet Sci. 2022 Sep 22;9:979548. doi: 10.3389/fvets.2022.979548 (PMC9536149; doi:10.3389/fvets.2022.979548)
Supplement: Supplementary file 5 [file Data_Sheet_2.PDF]

2022 Back Yard Chickens study

Start of Block: Default Question Block

Q1
You are being invited to take part in a research study about back yard (residential, non-commercial) chicken flocks in Vermont. Even if you do not currently or have never had back yard chickens, we would greatly appreciate your answer to the question below: *Do you currently, or have you had in the past year, a back yard chicken flock?*
We are attempting to determine about how many back yard chicken flocks are in Vermont and your response will help with this.

This study is being conducted by Andrea Etter at the University of Vermont with funding from the U.S. Department of Agriculture's Extension Service. The purpose of this study is to determine how many back yard chicken flocks are in Vermont and to collect information on health safety practices in order to understand factors that may influence the prevalence of Salmonella in backyard flocks of chickens in Vermont.

Study Procedures- If you choose to take part in the study, you will be asked to answer an online survey that will take about ten minutes of your time. The survey is completely confidential.

Benefits- As a participant in this research study, there is unlikely to be any direct benefit for you; however, information from this study may benefit other people now or in the future.
Risks- We will protect the information we collect from you during the study. We do not report individual responses and we store survey results on secure, password-protected computer servers.
Costs- There is no cost to you to participate in this research study, and you will not be paid for taking part in this study.
Confidentiality- All information collected is completely confidential. Your answers are not associated to your contact information and only the research team has access to the data we collect.
Voluntary Participation/Withdrawal- Taking part in this study is voluntary. You are free to not answer any questions or withdraw at any time. You may choose not to take part in this study, or if you decide to take part, you can change your mind later and withdraw from the study. If you withdraw without completing the survey, your data will be excluded from the study.
Questions- If you have any questions about this study now or in the future, you may contact (Andrea Etter) at the following phone number: (802)-656-0541 or via email at andrea.etter@uvm.edu with the subject line "back yard chicken study". If you have questions or concerns about your rights as a research participant you may contact the University of Vermont's Research Protections Office at (802) 656-5040.
You may print this information sheet for your records before continuing.

Respondents over the age of 18 years may participate. Are you over 18?

○ Yes (1)

○ No (2)

Q2 Do you currently have a backyard flock of chickens or have you had a flock in the last year?

○ Yes (1)

○ No (2)

Q3 And please select the Vermont town you currently reside in.

▼ ADDISON (1) ... Refuse (257)

End of Block: Default Question Block

Start of Block: Block 2

*

Q4 About how many chickens do you have now, or have you had on average over the past year?

____

Q5 Do you have chickens for: (Select any that apply)

- ☐ Eggs (1)
- ☐ Meat (2)
- ☐ Pets (3)
- ☐ Some other reason (Please describe) (4) ________________

Q6 What are your primary reasons for keeping chickens? (Select any that apply)

- ☐ Eggs/Meat tastes better than store bought (1)
- ☐ Pest control, like ticks (2)
- ☐ Food independence (3)
- ☐ Fun (4)
- ☐ Some other reason (Please describe) (5) ________________

Q7 How are your chickens primarily housed? (Besides their coop)

- ○ Coop with free range beyond a penned area (1)
- ○ Coop with a fixed penned in area (2)
- ○ Indoor housing only (inside a building, coop, etc.) (3)
- ○ Coop with a movable penned in area (mobile chicken unit, etc.) (4)
- ○ Some other housing option (Please describe) (5) ________________

Q8 What are your chicken's food source(s)? (Select any that apply)

- ▢ Forage (natural ground area) (1)
- ▢ Commercial feed (purchased from a store) (2)
- ▢ Table scraps/food scraps (3)
- ▢ Another food source? (Please describe) (4) ____

Q9 Do you have any other animals? (Select any that apply)

- ▢ Dogs (1)
- ▢ Cats (2)
- ▢ Horses (3)
- ▢ Cattle (4)
- ▢ Sheep (5)
- ▢ Goats (6)
- ▢ Pigs (7)
- ▢ Other poultry (Please Describe) (8) ____
- ▢ Other animals (Please Describe) (9) ____

Q10 How do you treat your chickens if they are sick? (Select any that apply)

- ☐ Antibiotics/veterinary-prescribed medicines (1)
- ☐ Natural remedies (herbs, essential oils) (2)
- ☐ Home remedies not specifically "natural" (3)
- ☐ Put them down (4)
- ☐ Some other treatment (Please describe) (5) ____

Display This Question:

If Do you have chickens for: (Select any that apply) = Eggs

Q11 How do you typically process eggs from your backyard chickens?

- ○ Wash and Refrigerate (1)
- ○ Wash and do not Refrigerate (4)
- ○ Don't wash and do Refrigerate (2)
- ○ Don't wash and do not Refrigerate (3)

Q12 What habits do you follow when handling your chickens or their eggs? (Select any that apply)

- ☐ Wash hands after handling chickens (1)
- ☐ Wash hands after handling eggs (2)
- ☐ Wash hands after handling dirty eggs (3)
- ☐ Change shoes after walking in chicken area (4)
- ☐ Wear gloves when handling chickens/eggs (5)
- ☐ Wear a mask when cleaning the chicken coop (6)
- ☐ Avoid kissing birds (7)
- ☐ Avoid snuggling birds (8)
- ☐ Keep children from snuggling birds (9)
- ☐ Keep children from interacting with chickens (10)
- ☐ Another habit? (Please describe) (11) ____

Q13 How often do children physically interact (petting, picking up, etc.) with your chickens? (Besides getting eggs)

- ○ Never (1)
- ○ Rarely (2)
- ○ Often (3)
- ○ Always (4)

End of Block: Block 2

Start of Block: Block 3

Q14
Now we have a few questions about your opinions of salmonella and chickens. Please provide your answers for the following health statements.

In your opinion, can chickens carry Salmonella while appearing healthy?

○ Yes (1)

○ No (2)

○ I don't know (3)

Q15 Can an egg have Salmonella on the inside, outside or both?

○ Inside (1)

○ Both inside or outside (2)

○ Outside (3)

○ I don't know (4)

Q16 In your opinion, are backyard flocks more or less likely to have Salmonella than commercial flocks?

○ More Likely (1)

○ Equally Likely (2)

○ Less Likely (3)

○ I don't know (4)

Q17 In your opinion, are eggs from backyard flocks more or less likely to contain Salmonella than eggs from the store?

- More likely to contain salmonella than eggs from the store (1)
- Equally likely to contain salmonella (2)
- Less likely to contain salmonella than eggs from the store (3)
- I don't know (4)

Q18 In your opinion, are eggs from your chickens more or less likely to contain Salmonella than eggs from the store?

- More likely to contain salmonella than eggs from the store (1)
- Equally likely to contain salmonella (2)
- Less likely to contain salmonella than eggs from the store (3)
- I don't know (4)

Q19 Have you ever taken a food safety or food microbiology class or taken part in a training that included information on food safety?

- I have taken a relevant class (1)
- I have participated in a relevant training (2)
- I have not taken a relevant class or training (3)

Q20 How do you get information about raising chickens?

- ☐ University Extension Education materials, website, trainings (1)
- ☐ Veterinarians (2)
- ☐ Books about raising chickens (3)
- ☐ Facebook or other social media sources (4)
- ☐ Instructional/informational Websites, Youtube, etc. (5)
- ☐ Magazines (6)
- ☐ Talking with others who raise chickens (7)
- ☐ Some other source? (8) ____

End of Block: Block 3

Start of Block: Block 4

Q21
Thank you for your responses. The survey is nearly complete.

We just have a few demographic questions to help us better understand who in Vermont is raising backyard chickens.

A reminder that your answers are completely confidential and we only report the total results.

Do you have people in your household who are under 18 years of age?

- ○ Yes (1)
- ○ No (2)

Q27 Select the option that best describes the neighborhood where you have chickens.

- ○ Rural countryside (1)
- ○ Suburban village or housing development (2)
- ○ Urban city or town (3)

Q22 Please write in your gender identification.

____

Q23 Please select the category that includes your age.

- ○ 18-24 (1)
- ○ 25-34 (2)
- ○ 35-44 (3)
- ○ 45-54 (4)
- ○ 55-64 (5)
- ○ 65 or over (6)

Q24 Please select the category that best represents your total household income after taxes in 2020.

- ○ $25,000 or less (1)
- ○ $25,000-$45,000 (2)
- ○ $45,000- $65,000 (3)
- ○ $65,000-$85,000 (4)
- ○ $85,000 or more (5)

Q25 What is the highest level of education you've completed?

- ○ Less than High School/Secondary School (no diploma, certificate, etc.) (1)
- ○ High School degree & Equivalent (2)
- ○ Some College or University (No degree, certificate) (3)
- ○ College, University, Technical degree, Certificate, etc. (4)
- ○ Advanced degree, Graduate degree (6)

Q26 Finally, have you had diarrheal symptoms lasting more than a day in the last year, or since you began raising chickens

- ○ Yes (1)
- ○ No (2)

End of Block: Block 4

Start of Block: Exit Block
